# New biomarkers for early diagnosis of Lesch-Nyhan disease revealed by metabolic analysis on a large cohort of patients

**DOI:** 10.1186/s13023-014-0219-0

**Published:** 2015-01-23

**Authors:** Irène Ceballos-Picot, Aurélia Le Dantec, Anaïs Brassier, Jean-Philippe Jaïs, Morgan Ledroit, Julie Cahu, Hang-Korng Ea, Bertrand Daignan-Fornier, Benoît Pinson

**Affiliations:** Laboratoire de Biochimie métabolomique et protéomique, Hôpital Necker-Enfants Malades, AP-HP, 149 rue de Sèvres, Paris, 75015 France; Université Paris Descartes Sorbonne Paris Cité, 15 rue de l’Ecole de Médecine, Paris, 75006 France; Centre de référence “Maladies Métaboliques Héréditaires de l’enfant à l’adulte” Hôpital Necker-Enfants Malades, AP-HP, 149 rue de Sèvres, Paris, 75015 France; Université de Bordeaux, IBGC UMR 5095, 1 rue Camille Saint-Saëns, Bordeaux, F-33077 France; Institut de Biochimie et Génétique Cellulaires, CNRS UMR 5095 1 rue C. Saint-Saëns CS 61390 F-33077, Bordeaux, France; Service de Biostatistique, Hôpital Necker-Enfants Malades, AP-HP, 149 rue de Sèvres, Paris, 75015 France; Université Paris 7 Denis Diderot, Hôpital Lariboisière, Centre Viggo Petersen, Inserm UMR 1132 (Ex-606), 2 rue Ambroise Paré, Paris, 75010 France

**Keywords:** HGprt, Hypoxanthine-guanine phosphoribosyltransferase, Deficiency, Genotype, Metabolome, Lesch-Nyhan disease, Variants, AICAR

## Abstract

**Background:**

Lesch-Nyhan disease is a rare X-linked neurodevelopemental metabolic disorder caused by a wide variety of mutations in the *HPRT1* gene leading to a deficiency of the purine recycling enzyme hypoxanthine-guanine phosphoribosyltransferase (HGprt). The residual HGprt activity correlates with the various phenotypes of Lesch-Nyhan (LN) patients and in particular with the different degree of neurobehavioral disturbances. The prevalence of this disease is considered to be underestimated due to large heterogeneity of its clinical symptoms and the difficulty of diagnosing of the less severe forms of the disease. We therefore searched for metabolic changes that would facilitate an early diagnosis and give potential clues on the disease pathogenesis and potential therapeutic approaches.

**Methods:**

Lesch-Nyhan patients were diagnosed using HGprt enzymatic assay in red blood cells and identification of the causal *HPRT1* gene mutations. These patients were subsequently classified into the three main phenotypic subgroups ranging from patients with only hyperuricemia to individuals presenting motor dysfunction, cognitive disability and self-injurious behavior. Metabolites from the three classes of patients were analyzed and quantified by High Performance Ionic Chromatography and biomarkers of HGprt deficiency were then validated by statistical analyses.

**Results:**

A cohort of 139 patients, from 112 families, diagnosed using HGprt enzymatic assay in red blood cells, was studied. 98 displayed LN full phenotype (86 families) and 41 (26 families) had attenuated clinical phenotypes. Genotype/phenotype correlations show that LN full phenotype was correlated to genetic alterations resulting in null enzyme function, while variant phenotypes are often associated with missense mutations allowing some residual HGprt activity. Analysis of metabolites extracted from red blood cells from 56 LN patients revealed strong variations specific to HGprt deficiency for six metabolites (AICAR mono- and tri-phosphate, nicotinamide, nicotinic acid, ATP and Succinyl-AMP) as compared to controls including hyperuricemic patients without HGprt deficiency.

**Conclusions:**

A highly significant correlation between six metabolites and the HGprt deficiency was established, each of them providing an easily measurable marker of the disease. Their combination strongly increases the probability of an early and reliable diagnosis for HGprt deficiency.

**Electronic supplementary material:**

The online version of this article (doi:10.1186/s13023-014-0219-0) contains supplementary material, which is available to authorized users.

## Background

The ubiquitous distribution of purine derivatives in human tissues and the high number of cellular functions in which these metabolites are involved explain why purine metabolism impairments lead to so many various diseases, *i.e.* >35 genetic pathologies are associated to purine metabolism genes (see [1] for review). The early recognition of these patients is required because of the progressive, irreversible and devastating consequences of these deficiencies [2]. A lot of these purine-associated pathologies share neurological, muscular, hematological and immunological symptoms. These common symptoms are most likely the consequence of nucleotide depletion and/or accumulation of toxic intermediates altering various biological functions, many of these deleterious effects taking place during embryonic development. Yet, the molecular mechanisms leading to these alterations are largely unknown and remain to be identified.

**Figure 1:**
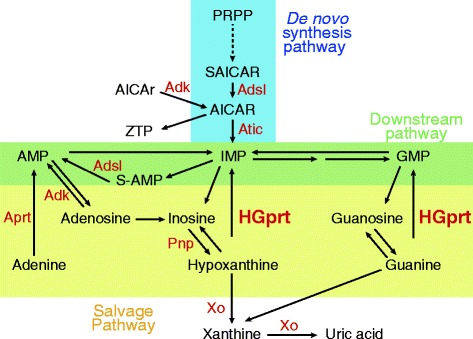
**Schematic representation of the human** ***de novo*** **, downstream and salvage purine pathways**. AICAr : 5-Amino-imidazole-4-carboxamide-1-β-D-ribofuranoside ; AICAR: AICAr 5’-monophosphate. AMP: Adenosine 5’-monophosphate; GMP: Guanosine 5’-monophosphate; IMP: Inosine 5’-monophosphate. PRPP: 5-phosphoribosyl-1-pyrophosphate. S-AMP: Succinyl-AMP. ZTP: AICAr 5’-triphosphate. Enzymes (in red): Adk: adenosine kinase; Adsl: Adenylosuccinate lyase; Atic: AICAR transformylase IMP cyclohydrolase; Aprt: Adenine phosphoribosyl Transferase; HGprt: Hypoxanthine Guanine phosphoribosyl Transferase; Pnp: Purine nucleoside phosphorylase Xo: Xanthine oxydoreductase.

Among purine-metabolism pathologies, the Lesch-Nyhan (LN) disease is a rare X-linked genetic disease, characterized in the most severe form by overproduction of uric acid, gout, severe motor disability, neurological deficiency and self-injurious behavior [3-5]. Milder forms of the disease, named Lesch-Nyhan Variants (LNV), exhibit less pronounced neurological and/or motor impairments and no self-injurious behavior [6-10]. A single mutated gene, *HPRT1,* is responsible for the LN pathology*. HPRT1* encodes the Hypoxanthine/Guanine phosphorybosyl transferase enzyme HGprt involved in two steps of the purine salvage pathway, *i.e.* conversion of hypoxanthine and guanine to inosine monophosphate (IMP) and guanosine monophosphate (GMP), respectively (Figure 1). The mutations are highly heterogeneous, with more than 400 different mutations already documented (http://www.lesch-nyhan.org/en/research/mutations-database/). Depending on the mutation, the enzyme exhibits none or residual enzymatic activity. Residual activity correlates with the severity of symptoms and in particular with the degree of neurological disturbances [3,11]. Hence, a phenotypic classification in three groups has now been accepted [3,4,9]. Lesch-Nyhan Disease (LND) patients display neurological deficiencies and self-injurious behaviors; they usually have undetectable HGprt activity. A second set of patients with various degrees of neuromuscular symptoms but no self-injurious behavior were grouped in HND (HGprt-related Neurological Dysfunction), they typically have a residual HGprt activity in live fibroblast assay. Finally, a third group of patients presenting no neurobehavioral disturbances and symptoms secondary to hyperuricemia only were classified as HRH (HGprt-Related Hyperuricemia) and generally have an enzymatic activity above 10%. Despite this correlation between enzymatic activity in live fibroblast and neurological disturbances, the underlying molecular mechanisms responsible for neurobehavioral troubles remain unknown. HGprt deficiency might affect homeostasis of purine metabolites, some of which play critical roles in neuronal differentiation and function and are toxic for the brain. Studies have shown that neurobehavioral syndrome is linked to reduction of dopamine in the basal ganglia [12] and demonstrated that HGprt deficiency is accompanied by deregulation of important pathways involved in the development of dopaminergic neurons [13-15]. The lack of a functional purine salvage pathway causes purine limitation in both undifferentiated and differentiated cells, as well as profound loss of dopamine content [16]. These results imply an unknown mechanism by which intracellular purine level modulates dopamine level.

In this study, we took advantage of a large cohort of 139 French patients to statistically evaluate the relationship between phenotype/genotype and purine, pyrimidine and pyridine content of red blood cells. Our aim was first to identify new biological markers to facilitate diagnosis of Lesch-Nyhan patients and second to provide clues on future therapy quests.

## Methods

### Patients and their classification

From 1980 to 2014, 139 patients from 112 families were diagnosed in our laboratory (I.C.P) using HGprt enzymatic assay in red blood cells. Clinical examination and HGprt dosage revealed 98 patients with the severe LND phenotype (86 families) and 41 LN variants (26 families) with attenuated symptoms. Lesch-Nyhan patients were thereafter classified according to prior validated phenotypic classification [4,9,17,18]. Briefly, in this cohort, 98 LND patients (in 86 families) with a full phenotype presented overproduction of uric acid, gout, severe motor disability, neurological deficiency and self-injurious behavior. Among LN variants, those presenting varying degree of neuromuscular symptoms but no self-injurious behavior (26 patients in 18 families) were grouped as HND, while 14 patients in 8 families with no neurobehavioral disturbances were grouped as HRH.

### Statement of ethical approval

The patient DNA and red blood cells collections used in this study were declared (N° DC-2009-955) at the “Plateforme de Ressources Biologiques” from Necker University Hospital (Paris; France) primarily for diagnosis and re-qualified for research purpose with the written consent of each patient or their parents.

### Mutation of *HPRT1*

The sequencing analysis was performed on 85 patients from 65 families among the 112 families of the French registry. A legal informed consent was obtained from all patients. Molecular analysis of the *HPRT1* gene was performed on genomic DNA from LND (n = 54 in 47 families), HND (n = 19 in 12 families) and HRH (n = 12 in 6 families) patients isolated from whole blood, as previously described by [7]. Briefly, the PCR primers used for exons 1–9 allowed genomic sequence analyses of both intron and exon segments involved in splice sequence mutations. All 9 exons of the *HPRT1* gene were amplified on 8 separate DNA fragments, with different lengths, using the kit AccuPrime GC rich for exon 1 and the kit Platinum Pfx for exons 2–9 (Invitrogen, Carlsbad, CA). The amplified fragments were purified using Illumina Exostar (Illumina) and sequenced using the same primers.

### Metabolic analyses

All metabolite extractions were performed on the blood samples used for the clinical diagnosis of patients. Metabolite extraction was achieved by boiling 100 μl of red blood cells in 5 ml of an ethanol/HEPES 10 mM pH 7.2 (3/1) solution for 3 min at 80°C. Samples were evaporated using a rotavapor apparatus (8 min, 65°C) and the dried residue was resuspended in 500 μl of MilliQ water. Insoluble particles were removed by centrifugation (21,000 g, 4°C, 1 hour) and the supernatant was ultra-filtrated on nanosep10K Omega (Pall). Metabolites were then separated on an ICS3000 chromatography station (Dionex, Sunnyvale, USA) using a carbopac PA1 column (250 × 2 mm; Dionex) with a 0.25 ml/min flow. Elution of metabolites was achieved with a Na-Acetate (NaAc) gradient in 50 mM NaOH as follows: elution was started at 50 mM NaAc for 2 min, rising up to 75 mM in 8 min, then to 100 mM in 25 min and finally to 350 mM in 30 min, followed by a step at 350 mM for 5 min, rising to 500 mM in 10 min, kept at this Na-Ac concentration for 5 min and finally raised up to 800 mM in 10 min followed by a step at this concentration for 20 min. The resin was then equilibrated at 50 mM NaAc for 15 min before injection of a new sample. Peaks were identified by their retention time and their UV spectrum signature (Diode Array Detector Ultimate 3000 RS, Dionex) and when necessary by co-injection with standards. When sufficient blood samples were available, metabolic analyses were performed on two independent metabolite extractions from patient’s red blood cells. The following metabolite samples were obtained and analyzed: 32 samples from asymptomatic patients (48 independent extracts); 29 samples from LND patients (47 independent extracts); 15 samples from HND patients (23 independent extracts); 12 samples from HRH patients (21 independent extracts); 13 samples from non Lesch-Nyhan patients presenting hyperuricemia (25 independent extracts). Metabolite amounts were normalized to hemoglobin content in the red blood cell samples measured spectrophotometrically (546 nm) using the Drabkin’s colorimetric reagent (5 ml; Chem-Lab NV, Belgium) on 20 μl of red blood cells samples. Of note, the metabolic extractions were done on red blood cells kept frozen at −20°C since the blood sampling. Statistical analyses revealed no significant correlation between any metabolic variation and the duration of freezing.

### Statistical analysis

Qualitative data were described by the sample size and percentage of each variable class. For quantitative data, we used quartile based indices (median, first-third quartile, min-max) to summarize variables and Wilcoxon rank-sum test to perform group comparisons. To summarize informative value of each metabolite as a diagnostic biomarker, we computed the area under the curve of the Receiver Operating Curve (AUC-ROC) [19]. Briefly AUC-ROC evaluates the ability of a quantitative marker to discriminate between two groups (for example patients and controls), a value of 1 indicates a perfect discrimination and 0.5 a complete absence of discrimination. If we enumerate all possible patient-control pairs, AUC-ROC can also be interpreted as the percentage of correctly ordered pairs, *i.e.* pairs where the patient marker level is higher than the control one, if the marker is expected to be increased in patients, and reciprocally if the marker is expected to be higher in controls. All computations were performed with the R statistical package v2.5 (http://www.r-project.org/). For the ROC analyses, we used the R ROCR library [20]. All tests were performed using a bilateral formulation. P-values less than 5% were considered as statistically significant. All metabolic distributions and ROC curves were drawn using Graph-Pad Prism software.

## Results and discussion

### Genotype/Phenotype classification of full LND and LN Variants

**Figure 2:**
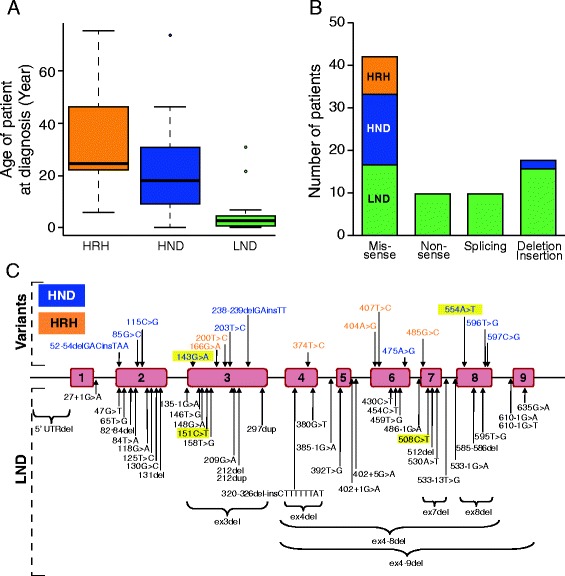
**Genotypic characterization of the French cohort of Lesch-Nyhan patients. A)** Average diagnosis age of the patient as function of symptoms severity. The median age is represented by the thick black line: HRH: HGprt-related Hyperuricemia (Median age 25 years old). HND: HGprt-related hyperuricemia with neuro-muscular dysfunction (Median age 18 years old). LND: full Lesch-Nyhan disease (Median age 3 years old). **B)** Distribution of mutation types throughout the *HPRT1* gene among the three groups of HGprt deficient patients. Black, blue and orange boxes represent mutations in LND, HND and HRH patients, respectively. **C)** Localization of the mutations on the *HPRT1* gene in the French cohort of LN patients. Briefly, mutations were identified by DNA exon and exon-intron junctions sequencing. In yellow boxes are the mutations found in more than one family.

The French cohort of Lesch-Nyhan patients was split into the three approved sub-groups [3] depending on the degree of neurological disturbances, from none to severe: HRH, HND, LND. It should be stressed that the average diagnosis age of the patient of the 3 groups varies significantly due to the obviousness of the most severe symptoms (Figure 2A). Sequencing analysis of the *HPRT1* gene revealed 61 different mutations among these 85 patients, spanning 65 families, 27 of which were novel. Of note, all these mutations including the 27 newly identified are listed in http://www.lesch-nyhan.org/en/research/mutations-database/. While the nature of the mutations was clearly not evenly distributed in the three groups (Figure 2B), we found that the location of the mutation was not a good prognostic marker of the severity of the disease (Figure 2C). The mutations were indeed scattered along the gene and no hot clusters were identified. This shows that multiple mutations of *HPRT1* can cause the unique Lesch-Nyhan disease. Within the LND group, 54 Lesch-Nyhan patients, spanning 47 families were analyzed. We found 45 different mutations distributed throughout the gene. For the Variants a total of 16 mutations were found in 19 HND patients (12 families) and in 12 HRH patients (6 families). Importantly, in LND, only 32% of mutations are missense, while 68% of the mutations were deletion, insertion, nonsense and splicing mutations (Figure 2B). Approximately half of the deletions were large intragenic deletions with loss of one or more exons. All mutations in introns gave rise to splicing error mutations and to LND. For the Variant forms HND and HRH, this tendency is completely reversed with a majority of missense mutations (88%) and the quasi-absence of deletion, nonsense and splicing mutations (Figure 2B). The difference in the type of mutations between LND and Variants suggests that mutations more susceptible to result in null enzymatic function or in abnormal protein conformation are more likely to cause the severe phenotype LND. By contrast, in the Variant forms, the missense mutations may allow some residual activity of the protein leading to a less severe phenotype.

### Identification of new biochemical markers for diagnosis of HGprt deficiency

**Figure 3:**
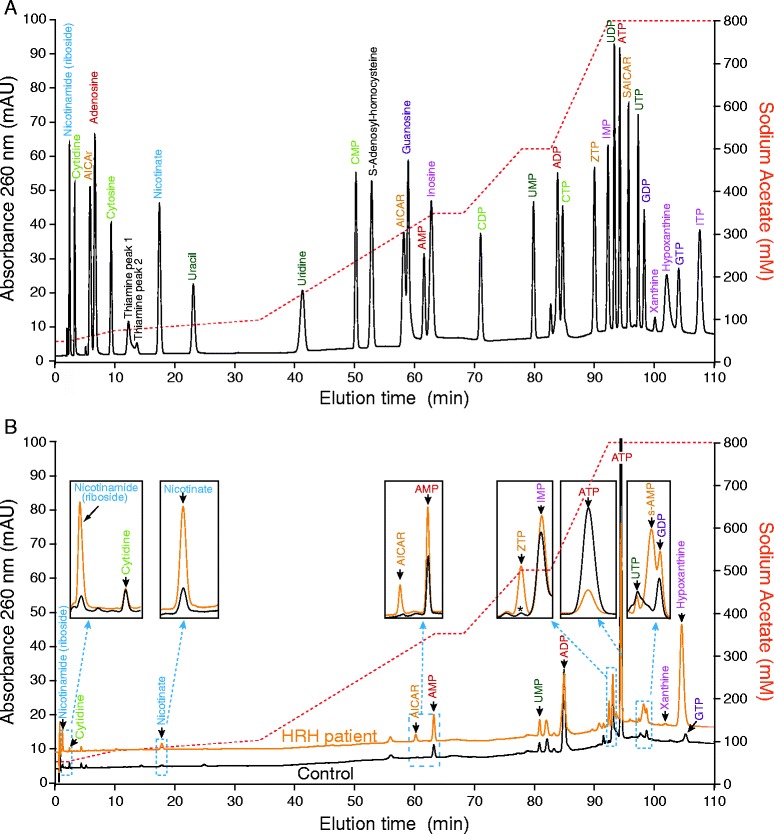
**Separation of standards (A) and red blood cell metabolic extracts (B) by ionic chromatography. A)** Standard metabolites profile was obtained by high performance ionic chromatography as described in Methods. Different colors refer to the different families of metabolites: blue: NAD(H) precursors; dark green: uridine derivatives; light green: cytidine derivatives; pink: inosine derivatives; purple: guanylic derivatives; red: adenylic derivatives; orange: AICAr derivatives and black: other detected metabolites. **B)** Representative chromatograms of red blood cells metabolic extracts from a control (black line) and a HRH patient (orange line). Insets represents zoom of the indicated regions. The Asterisk (ZTP inset) indicates an unidentified peak found in the control and that does not correspond to ZTP. The control and HRH extracts correspond to Control10 and HRH8 extracts (see Additional file 1: Table S1), respectively. **A B**, The red dashed line indicates the sodium acetate elution gradient. Nicotinamide (riboside) stands for the mix of nicotinamide and nicotinamide riboside, these two metabolites being not separated under our chromatographic conditions.

**Figure 4:**
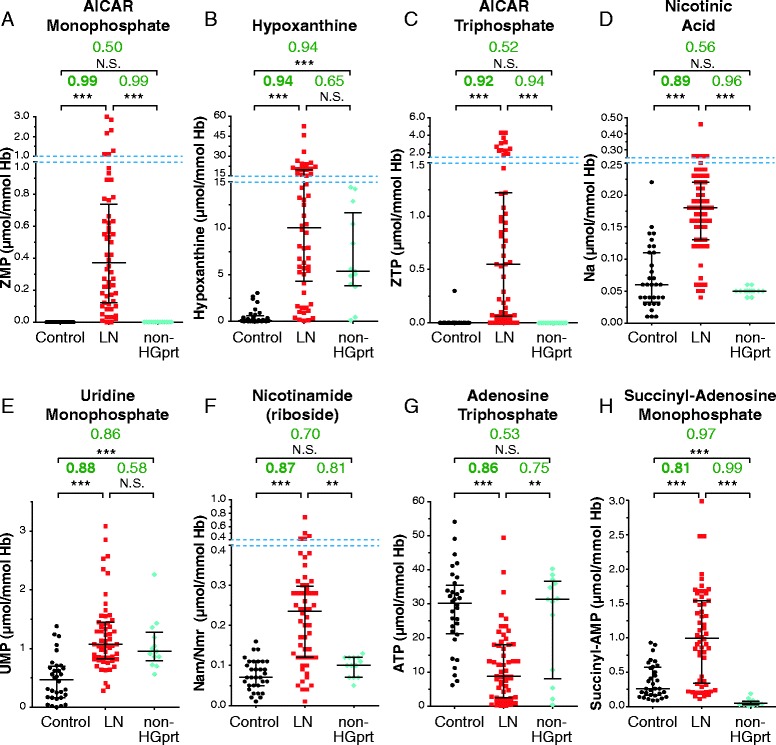
**Identification of the metabolites significantly changed in red cells of HGprt deficient patients. (A-H)** For all categories, each dot corresponds to the mean of metabolite content measured in independent red blood cell extracts. AUC values (green numbers) correspond to Area Under Curves values deduced from ROC (Receiver Operating Curves) analyses (Additional file 1: Figure S1) performed as described in Methods. p-values were obtained from a Mann–Whitney–Wilcoxon test. NS: non-statistically different = p-value > 10^−1^; *: p-value < 10^−2^; **: p-value < 10^−3^ and ***: p-value < 10^−4^. Control: healthy patients (black circles); LN: Lesch-Nyhan patients (HRH + HND + LND; Red squares); Non-HGprt: non HGprt-deficient patients with hyperuricemia (blue diamonds). Nicotinamide (riboside) stand for the mix of nicotinamide and nicotinamide riboside, these two metabolites being not separated under our chromatographic conditions. Raw data are presented in Additional file 1: Table S1. Dashed blue lines indicate scale breaks.

Blood samples from patients and controls were used to measure intracellular metabolite content in red blood cells. Because LND affects purine metabolism, we monitored the level of purine nucleotides, nucleosides and bases. In the same separation experiment we also measured pyrimidine nucleotides, nucleosides and bases as well as NAD(H) (Nicotinamide adenine di-nucleotide) and their precursors (Figure 3A). A comparison of representative chromatograms obtained with red blood cell extracts from a control and a HRH patient is presented in Figure 3B. Of note, GMP one of the products of HGprt (Figure 1) is not detectable in any control or Lesch-Nyhan patient red blood extracts. This observation is consistent with a previous one in which the presence of only the di- and tri-phosphate forms of guanylic nucleotides were detected in patient erythrocytes [21]. For each metabolite analyzed, statistical analyses of the data were conducted by drawing ROC (Receiving Operating Characteristic) curves and deducing the cognate AUC (Area Under Curve, see Methods for details). For eight metabolites the AUC were comprised between 0.8 and 1 (Figure 4A and Additional file 1: Figure S1) indicating a fair to excellent accuracy of the test in discriminating the groups being tested. Ten other metabolites presented some apparent differences between the control and LN patients, but statistical analyses revealed that for these metabolites AUC were between 0.80 and 0.5 and therefore had low or no significance (Additional file 1: Figure S2). Among the eight metabolites, significantly discriminating Lesch-Nyhan patients *versus* controls, are five purine derivatives (AICAR, hypoxanthine, ZTP (triphosphate form of AICAR), ATP and S-AMP), one pyrimidine (UMP) and precursors of NAD(H) (nicotinic acid and nicotinamide and/or nicotinamide riboside, these two last metabolites cannot be discriminated under our separation conditions). Importantly, some of these markers could reflect a more general hyperuricemia problem rather than being “specific” to HGprt deficiency. We therefore performed a similar metabolic analysis from blood samples from 13 hyperuricemic patients presenting normal HGprt activity (Figure 4 and Additional file 1: Table S1). In these patients two of the eight markers, *i.e.* hypoxanthine and UMP, were also accumulated in red blood cells (statistical results, Figure 4B and E), thus suggesting that hypoxanthine and UMP changes are more associated to hyperuricemia than to HGprt deficiency. In addition, while five of the thirteen hyperuricemic patients were treated with xanthine oxydoreductase inhibitor Allopurinol (Additional file 1: Table S1), we did not find any statistically significant difference on metabolites between treated and non-treated patients. From these analyses, we conclude that six metabolites significantly discriminate between HGprt deficient patients and healthy controls. We also performed a non parametric correlation analysis to determine is these six metabolites associated with HGprt deficiency could serve as biomarker of Lesch-Nyhan disease at any age of patients. For five of them (AICAR, ZTP, nicotinamide (riboside), ATP and S-AMP) we found no influence of age on the statistical relevance (p > 0.13, non-statistical significant correlation with age of patient for any of these metabolites) of using these metabolites as biomarkers for Lesch-Nyhan diagnosis. By contrast, for nicotinic acid, we found that strength of the biomarker significantly (p = 0.005, statistical significance **) decreases with age of the patient and is even not statistically significant for patients over 50 years old. Nevertheless, for these older patients the five other biomarkers are still fully statistically relevant for diagnosis of Lesch-Nyhan disease.

Among the six the metabolites, AICAR and ZTP, have very high AUC (0.99 and 0.92 respectively, Figure 4A and C and Additional file 1: Figure S1) and are therefore highly predictive biochemical markers. Accumulation of ZTP in LN patients has been reported previously on a small number of patients (5 LND + 1 LN variant) [21]. Our statistical analysis on a much larger cohort of LN patients definitely validates this biomarker. It should be stressed however that AICAR and ZTP accumulations have been reported in few patients with other purine metabolic disorders: purine nucleoside phosphorylase deficiency (Pnp; 1 patient), phosphorybosyl pyrophosphate (PRPP) synthetase over-activity (1 patient), and AICAR transformylase IMP cyclohydrolase deficiency (Atic, 1 patient) [21,22] and therefore could not be fully discriminative for diagnosis. Nonetheless, the combination of these two AICAR derivatives with the four other identified biomarkers with high AUC strongly increases the prognostic power. Importantly, our metabolic analysis was done on red blood cells which are prone to accumulate AICAR [23] and therefore amplifies this metabolic signal. Indeed, AICAR accumulation was not detected on patient fibroblasts (I.C.P. and B.P. unpublished results). It thus appears that red blood cells, which are easy to collect, are also providentially propitious to the use of these biomarkers. Of note, one could expect that alteration of HGprt activity in LN patients lead to a decrease in IMP pool, but we found no significant difference in IMP content between LN patients and healthy controls (Additional file 1: Figure S2). One possible explanation for this is that, in erythrocytes, AICAR is used as a precursor of a metabolic bypass that can replenish the IMP pool in a HGprt-deficient context (Figure 1) [24].

To address the role of HGprt in the pathogenesis of LND, several *in vitro* studies have been already performed comparing nucleotide metabolism in different cell types (lymphoblast and fibroblasts) from LND patients versus subjects with normal HGprt activity, or tissue/cell lines from *HPRT1* gene knock-out (KO) mice. The results were rather inconsistent [25-28]. Contributing factors to explain such variations have included culture conditions, unappreciated differences in growth rates and variability in precursor concentrations (e.g. nicotinamide) in different culture conditions [26,27,29]. Here, the analysis of erythrocytes, a single cell type directly sampled from patients, allowed to bypass these problems. Some of these earlier studies documented altered pyridine and purine nucleotide metabolite content in LND erythrocytes showing elevated NAD(H), as well as low GTP and ATP concentrations [30]. In addition, studies of NAD(H) metabolism on different tissues from HGprt gene knock-out (KO) mice revealed that NAD^+^ concentration was significantly increased in liver but not in brain or blood of the KO mice [31]. By contrast, NAD^+^ was found severely decreased in fibroblast from Lesch-Nyhan patients [29]. These results and ours demonstrate that changes in NAD(H) metabolism occur in response to HGprt deficiency, depending both on species and tissue type, however the physiopathological consequences remain to be explored.

### Correlation of metabolic profiles with clinical severity

**Figure 5:**
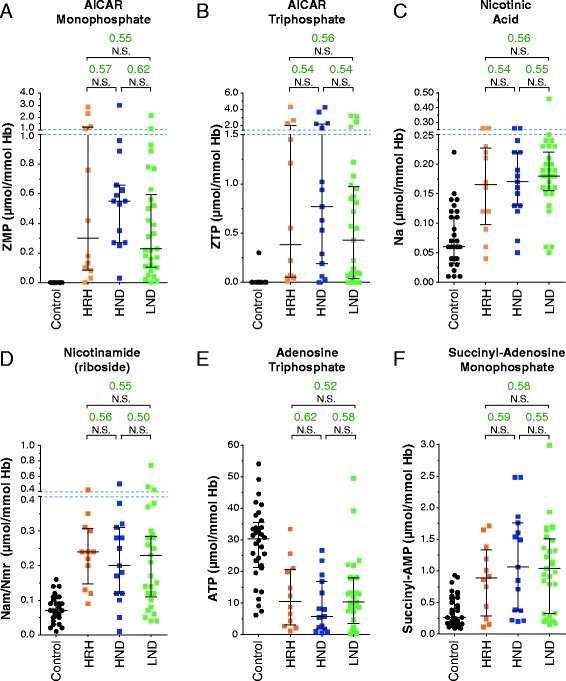
**Changes in identified biomarkers are not correlated with severity of the disease. (A-F)** For all categories, each dot corresponds to the mean of metabolite content measured in independent red blood cells extracts. AUC values (green numbers) correspond to Area Under Curves values deduced from ROC analyses performed as described in methods. p-values were obtained from a Mann–Whitney–Wilcoxon test: NS: non-statistically different = p-value > 10^−1^. Control: healthy patients (black circles); HRH: HGprt-related hyperuricemia (orange squares); HND: HGprt-related hyperuricemia with neuromuscular dysfunction (blue squares); LND: full Lesch-Nyhan disease (green squares).

Our results presented in the previous section established that the metabolic profile of patients significantly differed from that of controls and from that of non-LND gouty patients indicating that beyond the HGprt deficiency resides a more complex metabolic disease. We then investigated whether the six biochemical markers identified in this study could be used to assess the severity of the disease. We found that this was clearly not the case since statistical analyses showed no significant differences for the six biomarkers between the different classes of patients (Figure 5 A to F). However, it should be emphasized that for the two most severe forms of the disease (LND and HND) self-injurious behaviors and/or neuromuscular symptoms are easily diagnosed and the biomarkers in these cases could mostly be used for confirmation together with HGprt activity measurement. For the last group of patients suffering from hyperuricemia but presenting no neurobehavioral disturbances, HRH group, diagnosis often happens much later during life, frequently in adulthood (see Figure 2A). For these cases, the biochemical markers described here will be very useful to facilitate diagnosis at an early stage of the disease. The usefulness of these biomarkers would be further amplified if systematic search for hyperuricemia at birth was envisioned.

Why is there no clear correlation between the purine and pyrimidine metabolic profiles and the severity of the disease? A likely possibility is that the metabolic profiles, being derived from red blood cells of children or adults, does not fully reflect what happens in other cell types (especially neural tissues) and/or during embryonic development. A general question about the biomarkers identified in this study is whether they could be causative of some aspects of the pathology, as it was previously hypothesized concerning AICAR accumulation and the potential toxicity of this metabolite [32]. Massive accumulation of AICAR and its derivatives in Atic deficiency is associated with a devastating neurological disease involving profound mental retardation, epilepsy, dysmorphic features and congenital blindness [22]. In addition, AICAR is an inhibitor of the bi-functional enzyme Adenylosuccinate lyase (Adsl; Figure 1) and a deficiency of this enzyme also causes psychomotor retardation and autism in humans [33]. Indeed, the significant increase of succinyl-AMP (Figure 4H) in red blood cells of LN patients is in favor of a possible inhibition by AICAR of Adsl. AICAR, because of its structural similarity with AMP [34], is also a known activator of the AMP-activated protein kinase (AMPK), a homeostatic regulator of energy levels in the cell and influences the activity of a number of AMP-sensitive enzymes [35]. Accumulation of AICAR could have pleiotropic effects in the brain that could explain some of the neurological symptoms of Lesch-Nyhan patients. To establish a possible correlative link between AICAR accumulation and the severity of neurological symptoms in Lesch-Nyhan patients, metabolic analyses on cerebrospinal fluid would be more conclusive; however this biological material is not generally available. Of note, despite these difficulties, we could measure metabolic content in cerebrospinal fluid of few LND patients (8 patients) and healthy controls (6 patients). Even though purine derivatives were at very low concentration in these samples, we found some AICAR in its riboside form (AICAr) in Lesch-Nyhan patient cerebrospinal fluid but not in the controls (I.C-P. and B.P. unpublished results).

Our analysis on the LND patient erythrocytes also showed that the ATP concentration was significantly lower when compared to healthy control and/or gouty patients, as already observed by others [36]. A significant decrease in GTP concentration was also found (AUC 0.74, Additional file 1: Figure S2), thus confirming previous *in vitro* results obtained on human neuronal tissue culture [37]. The combined ATP and GTP depletion in erythrocytes could reflect the situation in the brain, which, like the erythrocyte, is largely dependent on salvage pathways to sustain its ATP and GTP levels. Because ATP is a downstream product of purine metabolism, HGprt deficiency results in energy limitation. Present knowledge assumes that defective dopaminergic transmission is an important cause for neurological deficit in LND [12], however the mechanisms responsible for dopamine loss in HGprt deficiency is still an enigma. Shortage of specific nucleotides may cause this abnormality through the inhibition of nigrostriatal axonal arborization at a developmental stage sensitive to nucleotide availability [13,14].

## Conclusion

We documented the molecular and biochemical analysis of Lesch-Nyhan disease in a large cohort of 139 patients from France belonging to 112 unrelated families. Sequence analysis of the *HPRT1* gene revealed that mutations were scattered along the gene and no hot clusters were identified. Importantly, within the most severe phenotypic group (LND) 68% of the mutations were deletion, insertion, nonsense and splicing mutations, mostly resulting in undetectable enzyme function. In Variant forms, HND and HRH, this tendency is completely reversed with a majority of missense mutations (88%), thus leading to residual HGprt activity. The effect of the *HPRT1* mutations on residual HGprt enzyme activity is a relevant factor contributing to disease phenotype. Diversity of inborn errors in nucleotide metabolism leading to a large spectrum of common neurodevelopemental symptoms makes the diagnosis difficult, especially for patients with the less severe forms of these various diseases. Our chromatographic method allowed us to identify six metabolites (dramatic increase for AICAR, ZTP, nicotinic acid, nicotinamide (riboside), S-AMP and severe ATP depletion) statistically fully associated to HGprt deficiency. These six metabolites define new specific biomarkers since they are not significantly modified in hyperuricemic patients without HGprt-deficiency. These new biochemical markers are easy to measure on red blood cell extracts and their combination increases the probability of an early and reliable diagnose of less severe forms of HGprt deficiency.

## Additional file

Additional file 1: Figure S1. Representation of Receiver Operating Curve used to calculate AUC (Area Under Curves). The Receiver Operating Curves were obtained as described in methods. “Sensitivity” (also called the true positive rate) represents the percentage of sick people who are correctly identified, while “Specificity” (also called the true negative rate) measures the proportion of healthy people correctly identified. A perfect metabolic marker would be described as 100% sensitivity (i.e. predicting all people from the sick group as sick) and 100% specificity (*i.e.* not predicting anyone from the control group as sick) thus leading to an AUC = 1. By contrast, a non discriminative metabolite would be found with an AUC close to 0.5. Dashed black diagonal represents the line of identity between the two groups. **Figure S2.** Other metabolic changes in red blood cells from HGprt deficient patients. For all categories, each dot corresponds to the mean of metabolite content measured in independent red blood cells extracts. AUC values (green numbers) correspond to Area Under Curves values deduced from ROC analyses performed as described in Methods. p-values were obtained from a Mann–Whitney–Wilcoxon test: NS: non-statistically different = p-value > 10^−1^; *****: p-value < 10^−2^; ******: p-value < 10^−3^ and *******: p-value < 10^−4^. Control: healthy patients (black circles); LN: Lesch-Nyhan patients (HRH + HND + LND; Red squares); Non-HGprt: non HGprt-deficient patients with hyperuricemia (blue diamonds). **Table S1.** Values are given in μmol metabolite/mmol Hemoglobin.
