# Supplementary figures and images for: New biomarkers for early diagnosis of Lesch-Nyhan disease revealed by metabolic analysis on a large cohort of patients
Source: Orphanet J Rare Dis. 2015 Jan 23;10:7. doi: 10.1186/s13023-014-0219-0 (PMC4320826; doi:10.1186/s13023-014-0219-0)

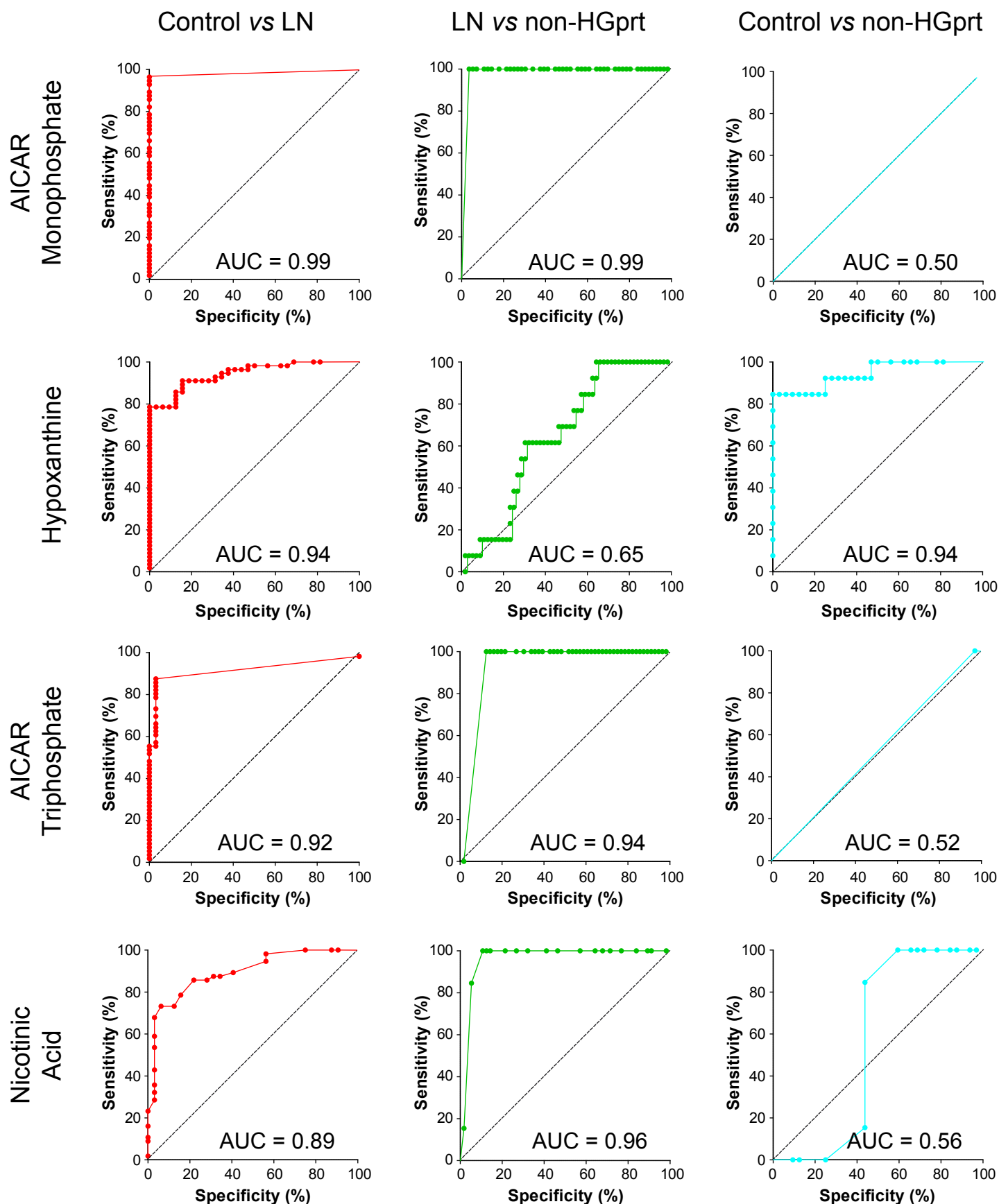

Ceballos-Picot *et al*  
Supplemental Figure 1 Part A

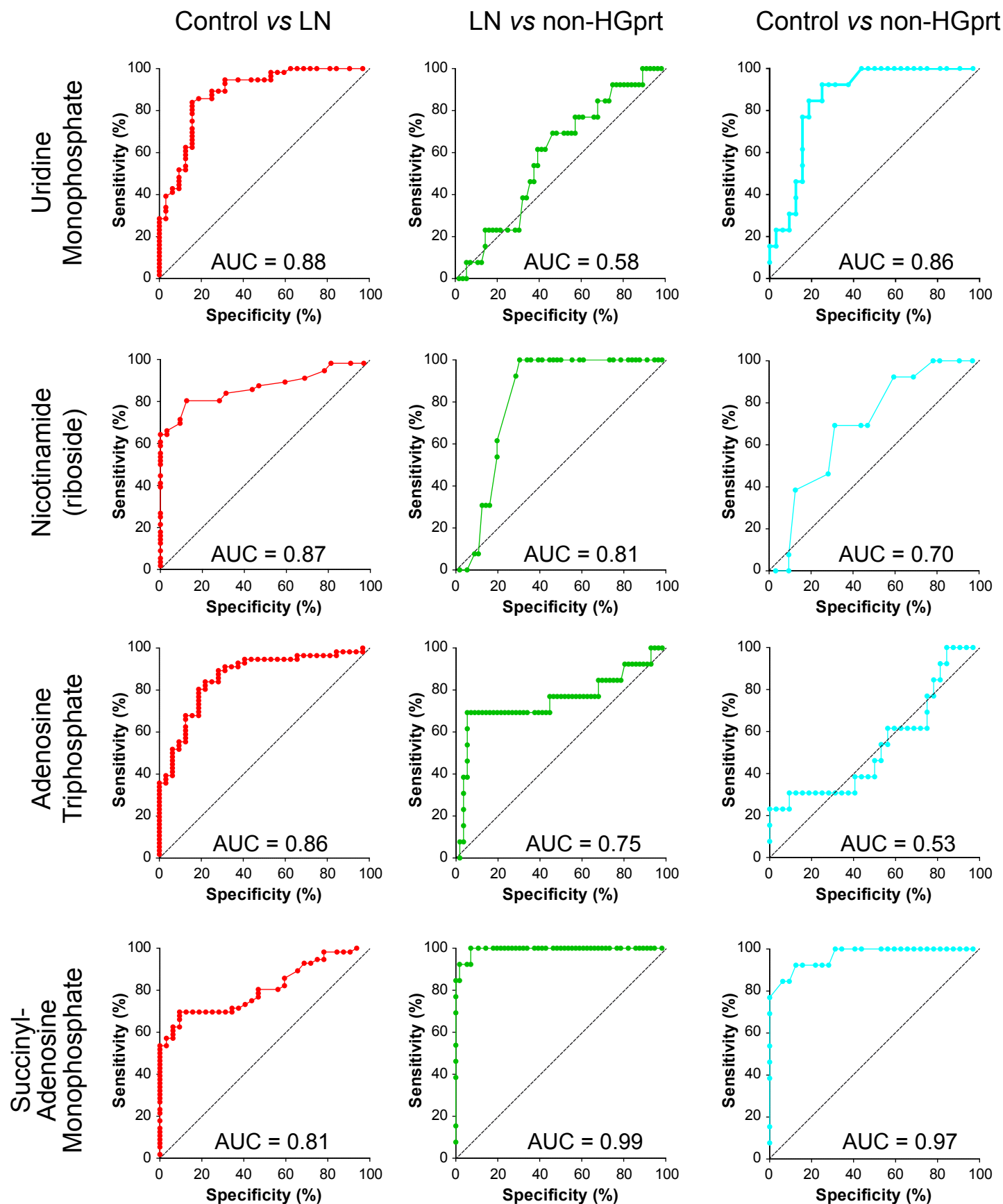

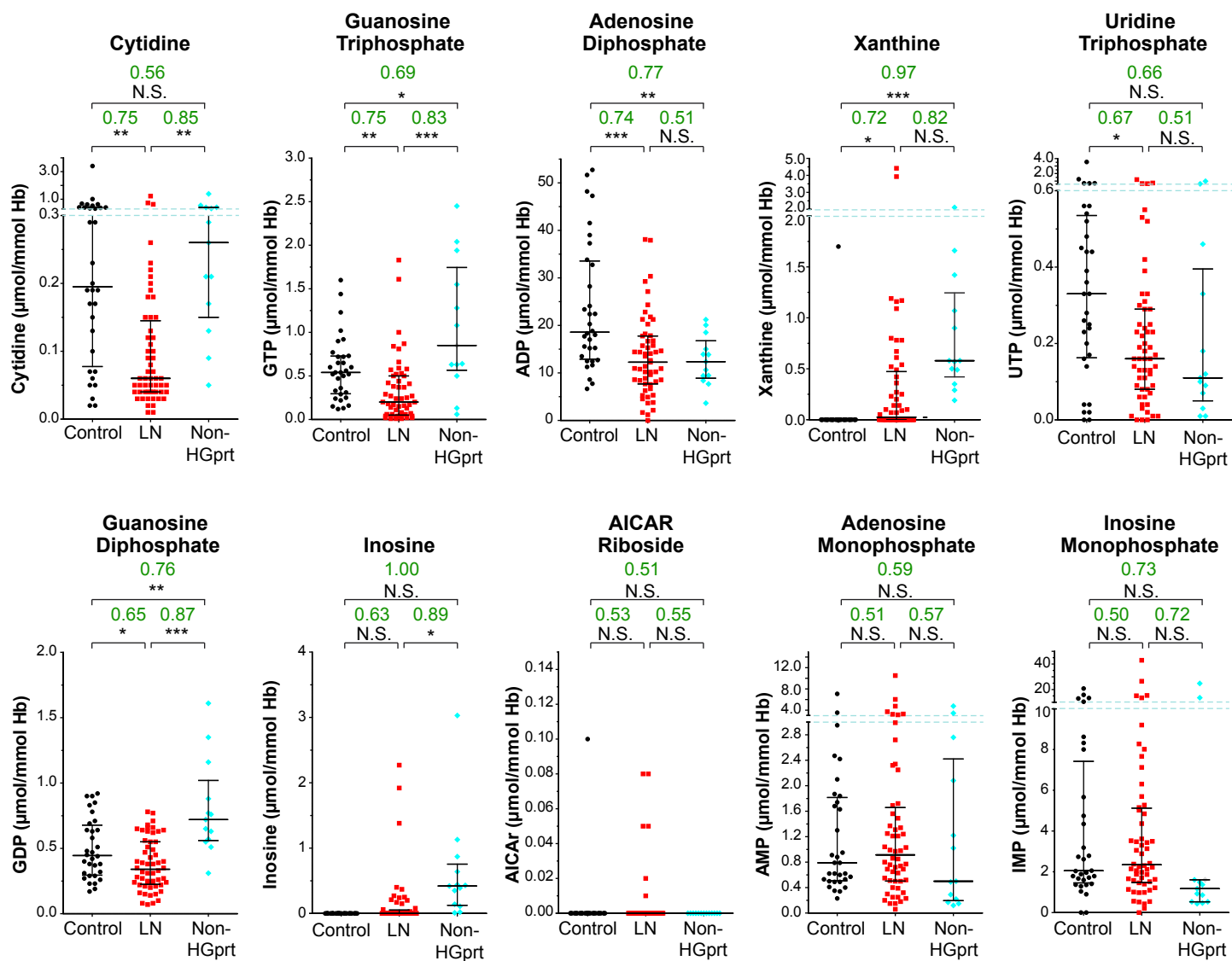

Ceballos-Picot *et al*  
Supplemental Figure 2

Supplement: Additional file 1: Figure S1. — Representation of Receiver Operating Curve used to calculate AUC (Area Under Curves). The Receiver Operating Curves were obtained as described in methods. “Sensitivity” (also called the true positive rate) represents the percentage of sick people who are correctly identified, while “Specificity” (also called the true negative rate) measures the proportion of healthy people correctly identified. A perfect metabolic marker would be described as 100% sensitivity (i.e. predicting all people from the sick group as sick) and 100% specificity (i.e. not predicting anyone from the control group as sick) thus leading to an AUC = 1. By contrast, a non discriminative metabolite would be found with an AUC close to 0.5. Dashed black diagonal represents the line of identity between the two groups. Figure S2. Other metabolic changes in red blood cells from HGprt deficient patients. For all categories, each dot corresponds to the mean of metabolite content measured in independent red blood cells extracts. AUC values (green numbers) correspond to Area Under Curves values deduced from ROC analyses performed as described in Methods. p-values were obtained from a Mann–Whitney–Wilcoxon test: NS: non-statistically different = p-value > 10−1; *: p-value < 10−2; **: p-value < 10−3 and ***: p-value < 10−4. Control: healthy patients (black circles); LN: Lesch-Nyhan patients (HRH + HND + LND; Red squares); Non-HGprt: non HGprt-deficient patients with hyperuricemia (blue diamonds). Table S1. Values are given in μmol metabolite/mmol Hemoglobin. [file 13023_2014_219_MOESM1_ESM.pdf]
